# Acetone compression improves lymph node yield and metastasis detection in colorectal cancer

**DOI:** 10.1007/s10585-023-10259-x

**Published:** 2024-01-04

**Authors:** Christina Schnoz, Katrin Schmid, Guacimara Ortega Sanchez, Sabina Schacher-Kaufmann, Michel Adamina, Georgios Peros, Dieter Erdin, Peter Karl Bode

**Affiliations:** 1 Department of Pathology, Kantonsspital Winterthur, Brauerstrasse 15, Winterthur, 8401 Switzerland; 2 Department of Medical Oncology and Hematology, Kantonsspital Winterthur, Brauerstrasse 15, Winterthur, 8401 Switzerland; 3 Department of Visceral and Thoracic Surgery, Kantonsspital Winterthur, Brauerstrasse 15, Winterthur, 8401 Switzerland

**Keywords:** Colorectal cancer, Lymphatic metastasis, Nodal staging, Acetone compression, Surgical pathology

## Abstract

Lymph node status is one of the most important prognostic factors in colorectal cancer, and accurate pathological nodal staging and detection of lymph node metastases is crucial for determination of post-operative management. Current guidelines, including the TNM staging system and European Society for Medical Oncology (ESMO) guidelines, recommend examination of at least 12 lymph nodes. However, identification of an adequate number of lymph nodes can be challenging, especially in the setting of neoadjuvant treatment, which may reduce nodal size. In this study, we investigated 384 colorectal cancer resections that were processed at our department of pathology between January 2012 and December 2022, in which the number of detected lymph nodes was less than 12 subsequent to conventional preparation of mesocolic fat tissue. By means of acetone compression, lymph node harvest increased significantly (*p* < 0.0001), and the intended number of ≥ 12 lymph nodes was achieved in 98% of resection specimens. The number of nodal positive cases increased significantly from n = 95 (24.7%) before versus n = 131 (34.1%) after acetone compression due to additionally identified lymph node metastases (*p* < 0.001). In 36 patients (9.4%) initially considered as nodal negative, acetone compression led to a staging adjustment to a nodal positive category and thereby drove a recommendation to offer post-operative therapy. In conclusion, acetone compression is a reliable and useful method implementable in routine surgical pathology for the retrieval of lymph nodes in colorectal cancer specimen, allowing for an adequate lymph node sampling and an increase in nodal staging reliability.

## Introduction

Colorectal cancer (CRC) is the third most common malignancy and the second leading cause of cancer-related death worldwide, with approx. 2 million new cases and almost 1 million deaths annually [1]. The main treatment of CRC is surgical resection of the primary tumor, combined with central lymphadenectomy [2, 3]. Lymphatic metastasis is considered a major prognostic factor for disease recurrence and survival in CRC patients [4]. Thus, lymph node staging remains one of the key criteria to determine post-operative management and to identify patients requiring chemotherapy and/or radiotherapy additional to surgery [5]. Radiotherapy in the pre-operative setting and chemotherapy, either applied pre- or post-operatively, are standard of care in many scenarios, depending on tumor localization and disease extent. In colon cancer, the current guidelines recommend post-operative chemotherapy in patients with stage III disease, defined by the presence of lymph node metastasis [6], independent of the number of lymphatic metastases [7, 8]. In stage II colon cancer (T3-T4, lymph nodes negative), post-operative chemotherapy is considered only in patients with certain high-risk features, such as a T4 category, lympho-vascular or perineural invasion, positive surgical margins, undifferentiated histology, surgery under emergency conditions, or inadequate sampled lymph nodes (i.e., number < 12) [9–13].

Compared to colon cancer, rectal carcinomas are approached therapeutically with certain differences. Due to the anatomical proximity to pelvic structures and a higher risk of local recurrence, neoadjuvant therapy is standard of care in locally advanced rectal cancer [14, 15]. However, the beneficial impact of post-operative therapy in rectal cancer remains controversial [16, 17]. Post-operative chemoradiotherapy is usually reserved for low-stage patients who have not received neoadjuvant treatment, and who are found to be higher stage after pathological review of the surgical specimen [18].

The N category in the TNM staging system for CRC is determined by the number of metastatic lymph nodes as N1 (1–3 metastatic lymph nodes) or N2 (≥ 4 metastatic lymph nodes) [6]. TNM staging system and other current guidelines such as European Society for Medical Oncology (ESMO) guidelines recommend examination of at least 12 lymph nodes to achieve an accurate staging [6, 11]. However, the number of lymph nodes retrieved from a surgical resection specimen is impacted by various factors, such as age, gender, tumor-specific characteristics (tumor size, localization, histology, grade), experience of surgeon and pathologist, and neoadjuvant treatment [19, 20]. In particular, pre-operative chemoradiotherapy in advanced stage rectal cancer has shown to diminish the number of lymph nodes retrieved [21, 22] due to nodal regression and reduction of lymph node size, which makes their macroscopic identification more difficult [23].

The conventional preparation method harvesting lymph nodes comprises manual palpation and dissection of lymph nodes within mesenteric fat. This may be a time-consuming procedure and depends on the experience of the individual examiner. Achieving the target of 12 lymph nodes at a minimum can be challenging in certain patients. In the past, several alternative techniques to maximize lymph node yield in colorectal resection specimens have been introduced [24]. Some of them, such as complete paraffin embedding of the entire mesorectal compartment, have proven favorable results in the detection of small lymph nodes, but are associated with considerable additional effort [25, 26]. Acetone compression is an alternate method that decreases mesenteric fat volume through dissolving fat in acetone and extracting dissolved fat with compression. Remaining tissue can be embedded and microscopically assessed for additional lymph nodes [27–29].

In this study, we analyzed our data from acetone compression in 384 colorectal cancer specimens in which the required number of 12 lymph nodes was not achieved priorly by conventional manual mesenteric fat dissection. The aim was to assess the extent to which acetone compression increased the number of detected lymph nodes. Furthermore, we addressed the issue of whether lymph node metastases identified by acetone compression led to a revision in final nodal staging.

## Materials and methods

### Data

Our department of pathology participates in the „Tumorzentrum Kantonsspital Winterthur“, which is a tumor center certified by the German Cancer Society (DKG). We investigated a total number of 384 colorectal cancer cases that were examined at our department of pathology between January 2012 and December 2022. In all these surgical specimens, the required number of twelve lymph nodes was initially not achieved by conventional manual dissection, and subsequent acetone compression of mesocolic and mesorectal fat was conducted. Only adenocarcinoma resections of colorectal cancer were included, other tumor types (i.e., gastrointestinal stromal tumor, carcinoid tumor) were excluded. The data analyzed was extracted from PathoWin+, the laboratory information system used at our institute comprising all patient’s data, clinical information, laboratory procedures performed and final pathology reports.

### Manual and acetone compression method

**Fig. 1:**
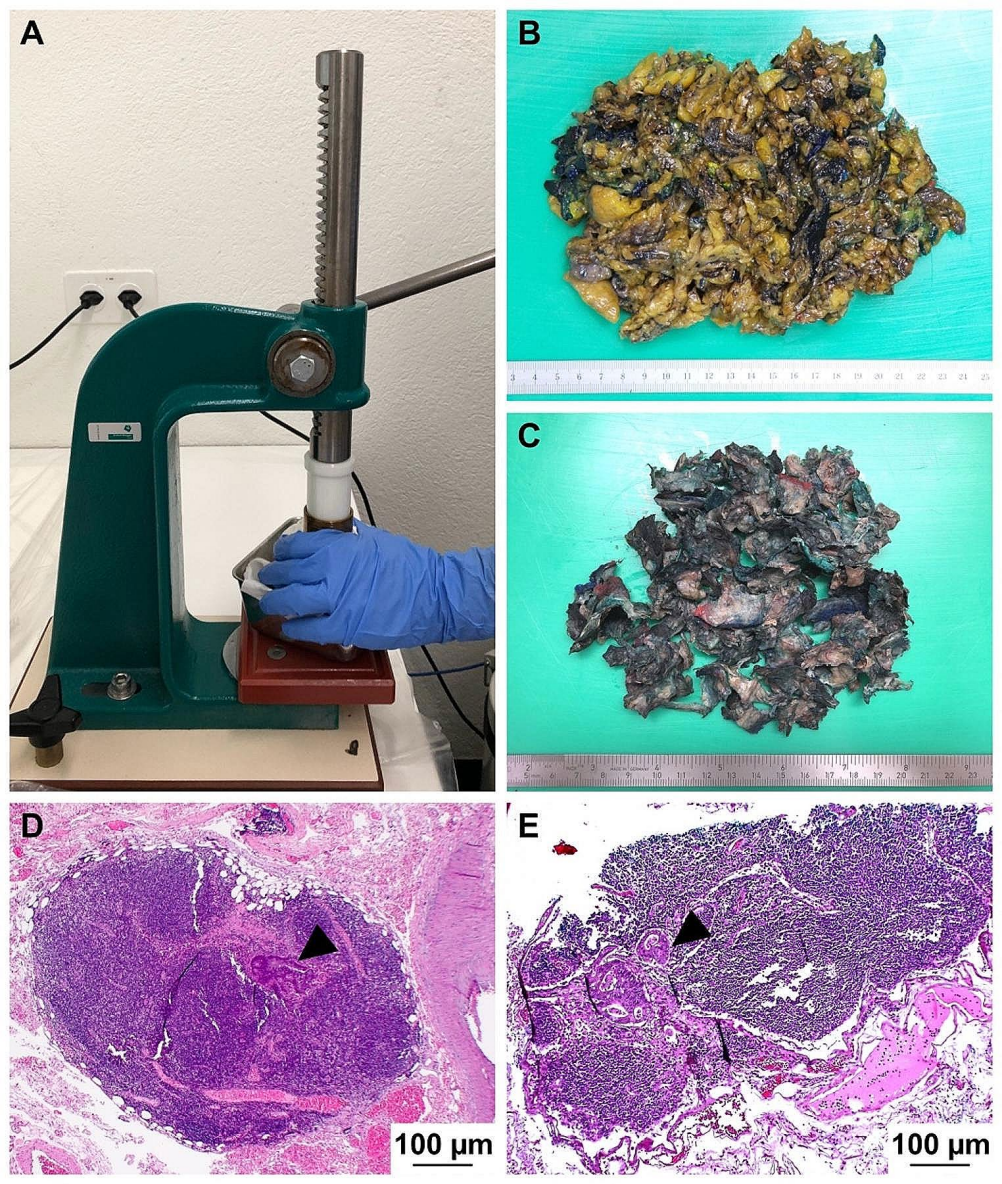
**Acetone compression method**. **(A)** After standard manual lymph node dissection, remnant adipose tissue was eluted in acetone for 24 h, with subsequent mechanical compression using a manual press [28]. **(B)** Sliced adipose tissue before acetone compression and **(C)** after fat removal applying acetone compression. Fat volume is significantly reduced and remaining tissue is entirely embedded in paraffin and submitted for microscopic examination. **(D, E)** Microscopically small lymph nodes of mesocolic fat tissue with micrometastases (< 2 mm) identified by acetone compression (HE stain)

As a first approach, lymph nodes of all surgical specimens were collected by conventional manual dissection of mesenteric fat tissue, comprising thin slicing and careful palpation of fatty tissue. As the required number of twelve lymph nodes was not identified macroscopically, subsequent acetone compression was performed. To this, all remaining fat tissued was fixed in formalin (formaldehyde 4%, Lobeck Chemie AG, Bad Zurzach, Switzerland) for at least 24 h. Tissue then was further perforated using a special needle stamp, followed by incubation in acetone (Artechemis AG, Zofingen, Switzerland), 1000 ml per 100 g of fat tissue for 24 h at room temperature. Next, tissue was placed on a paper and squeezed with a rolling pin in order to absorb the dissociated acetone-fat solution. Remaining tissue was then mechanically compressed with a manual stamp machine, and the obtained remnants were completely encapsulated for paraffin embedding and histology (Fig. 1).

### Statistical methods

Statistical analyses were performed using GraphPad Prism (GraphPad Software, Version 8.0.2). Normal distribution was tested using the Kolmogorow-Smirnow test. The differences in number of lymph nodes and metastases found per specimen before and after AC were compared using the Wilcoxon signed rank test. Comparison of nodal positive and nodal negative cases before and after AC was conducted using the McNemar’s test. Values were considered as significantly different when *p* < 0.05. Data are given as means +/- SD. The figures were created using GraphPad Prism.

## Results

### Patients’ characteristics

Overall, 384 cases of colorectal cancer were investigated. 255 patients were male (66%) and 129 were female (34%). Average age was 69.2 (± 12.2) years in male and 69.5 (± 13.0) years in female patients, respectively. 129 of tumors were located in the colon (34%), and 255 tumors in the rectum (66%). 186 patients (48%) received neoadjuvant treatment prior to surgery. Among those, the majority (n = 172) were treated with neoadjuvant chemoradiotherapy. 8 patients received pre-operative radiotherapy only, 4 patients received pre-operative chemotherapy only, and in 2 cases, the modality of neoadjuvant therapy could not be determined retrospectively. Patients’ characteristics and distribution of T stage are presented in Table 1.

**Table 1:** Patient and tumor characteristics

Feature	Number (Average)
**Gender**	
Male	255 (66%)
Female	129 (34%)
**Age (years)**	
Male	69.2 ± 12.2
Female	69.5 ± 13.0
**Tumor site**	
Colon	129 (34%)
Rectum	255 (66%)
**Neoadjuvant therapy**	
Yes	186 (48%)
No	198 (52%)
**T category**	
(y)pT0	29 (7.5%)
(y)pTis	4 (1%)
(y)pT1	49 (13%)
(y)pT2	91 (24%)
(y)pT3	152 (39.5%)
(y)pT4	59 (15%)
**No of lymph nodes**	
Before AC	4.8 ± 3.0
After AC	33.4 ± 14.3

### Total detected lymph nodes before vs. after acetone compression

**Fig. 2:**
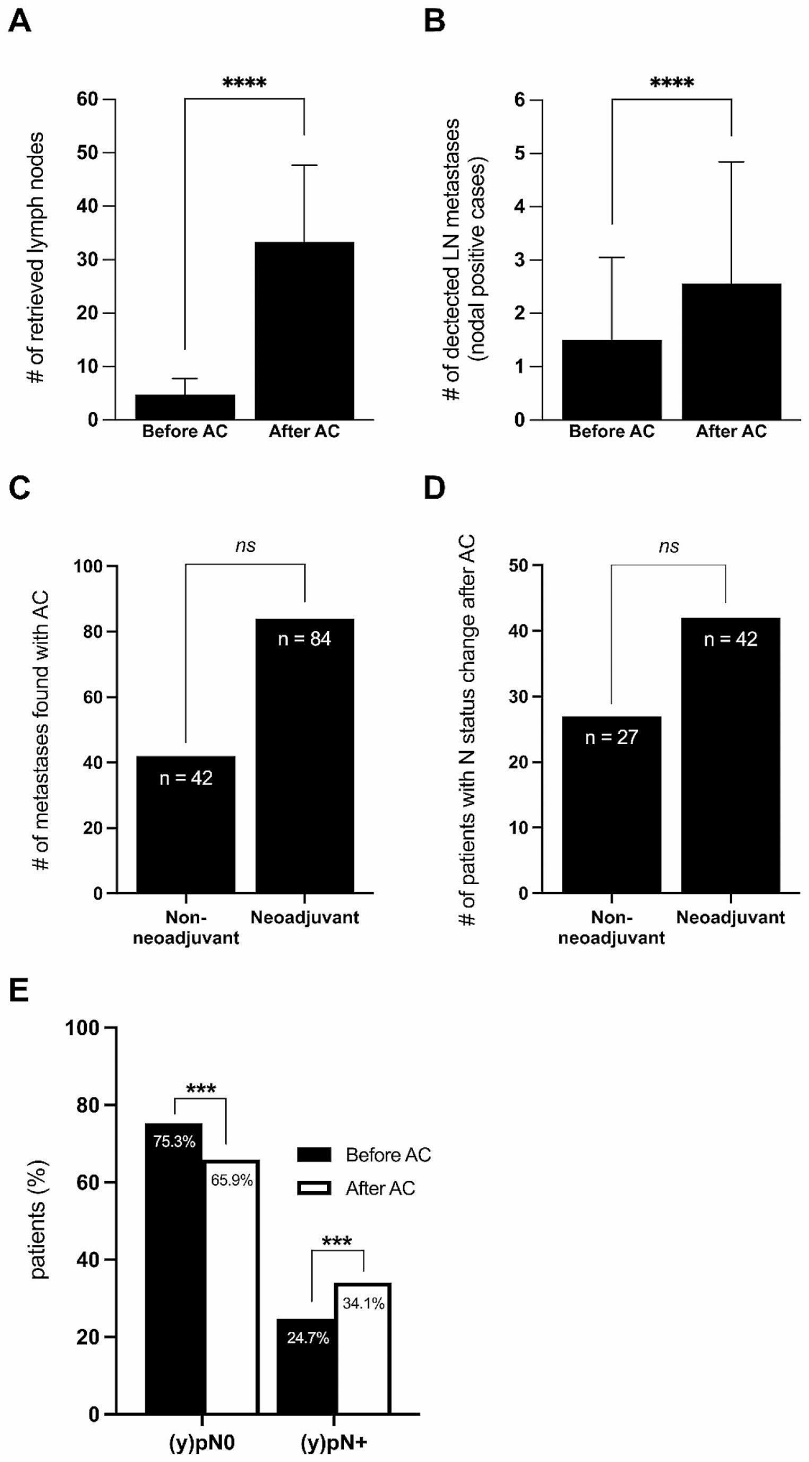
**Lymph node retrieval and metastasis identification through acetone compression**. **(A)** Average number of retrieved lymph nodes per specimen before and after AC. **(B)** Average number of detected metastatic lymph nodes per specimen within nodal positive cases before and after AC. **(C)** Total number of metastases identified by AC in non-neoadjuvant and neoadjuvant treated patients. **(D)** Number of non-neoadjuvant and neoadjuvant treated patients with a nodal staging adjustment due to AC. **(E)** Lymphatic metastases found by AC led to a significant increase in nodal positive cases and a significant decrease in nodal negative cases, respectively (*p* < 0.001)

Conventional manual lymph node dissection yielded a total of 1828 lymph nodes (average 4.8 ± 3.0 per case). With acetone clearance, a total of additional 10’978 lymph nodes were found, resulting in an average of 33.4 ± 14.3 per case. Thus, a significant increase in the number of detected LN was achieved by acetone compression (*p* < 0.0001) (Fig. 2A). In the majority of cases (n = 377, 98%), the intended number of ≥ 12 lymph nodes were successfully achieved, and only 7 resection specimens (2%) remained with less than 12 lymph nodes identified.

### Lymph node metastases before vs. after acetone compression

Manual lymph node preparation determined a total of 168 lymph node metastases. With AC, 126 additional metastases were identified. The mean number of lymph node metastases within nodal positive specimen increased significantly after acetone compression (1.5 ± 1.6 vs. 2.6 ± 2.3, *p* < 0.0001) (Fig. 2B).

Two third of metastases discovered by AC affected patients who underwent neoadjuvant treatment (n = 84, 66.7%), and one third of metastases found with AC were allocated to patients without neoadjuvant therapy (n = 42, 33.3%) (Fig. 2C). AC led to an adjustment of nodal staging in 42 neoadjuvant treated patients and in 27 patients without neoadjuvant therapy (Fig. 2D). However, these differences between the neoadjuvant and non-neoadjuvant treated groups did not reach statistical significance.

### Effect of acetone compression on N category and disease staging

N stage distribution before and after AC is presented in Table 2. Before AC, 289 patients (75.3%) were staged as nodal negative, whereas 95 patients (24.7%) were nodal positive. After AC, 253 patients (65.9%), remained nodal negative, whereas 131 cases (34.1%) were classified as nodal positive (*p* < 0.001). (Fig. 2E).

**Table 2:** Distribution of N category before and after acetone compression

N Category		
	Before AC	After AC
**(y)pN0**	289 (75.3%)	253 (65.9%)
**(y)pN1a**	47 (12.2%)	49 (12.8%)
**(y)pN1b**	21 (5.5%)	39 (10.1%)
**(y)pN1c**	11 (2.8%)	18 (4.7%)
**(y)pN2a**	15 (3.9%)	17 (4.4%)
**(y)pN2b**	1 (0.3%)	8 (2.1%)

**Fig. 3:**
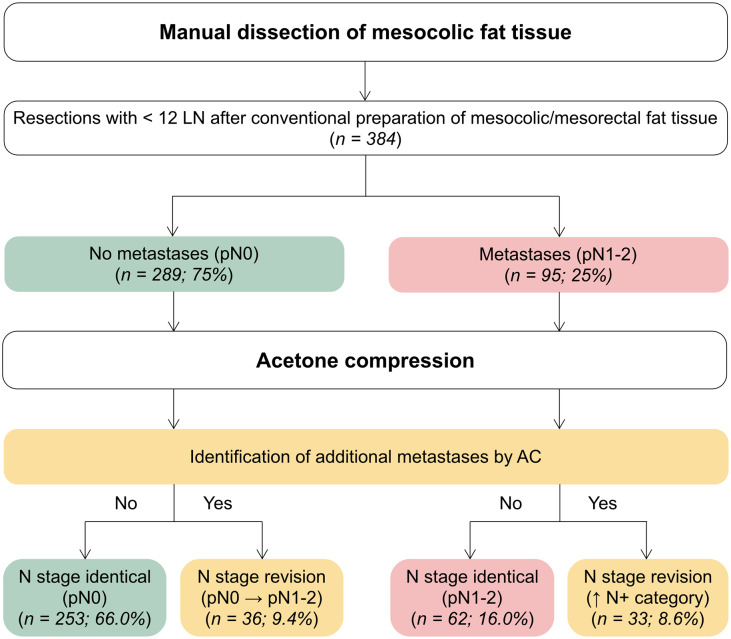
**Flow chart of lymph node exploration procedure, number of nodal positive cases and impact of acetone compression on N category**. Additional lymph node metastases identified by acetone compression led to a change in N category in 18.0% of cases. Thereof, 9.4% were reallocated from nodal negative to nodal positive due to LN metastases found by AC. 8.6% were already classified as nodal positive before AC, but AC led to an assignment to a higher N category

Overall, AC and its identification of additional LN metastases led to a modification in the nodal category in 69 patients (18.0% of total). Among those, 33 patients (8.6% of total) were already classified as nodal positive before AC, and additional LN metastases found with AC led to an allocation to a higher N category. 36 patients (9.4% of total) were initially considered as nodal negative and had to be re-classified as nodal positive due to LN metastases found with AC (Fig. 3). Of these, 21 patients (5.5% of total) had received neoadjuvant treatment.

## Discussion

Even though recommendations of an appropriate LN count vary, current guidelines recommend a minimum number of 12 LN for a reliable LN staging in CRC patients [11, 12, 30–32]. Our study of 384 cases proves that acetone compression method is a very effective technique to achieve a high lymph node yield, with a 6-7x increase in LN count. In almost every surgical specimen examined for this study, we succeeded in detecting 12 or more LN with AC, when prior manual LN dissection was not effective. Since LN metastases do not depend on lymph node size and may occur in macroscopically not detectable lymph nodes, examination of microscopically small LN is valuable [33, 34]. This is consistent with our observation, since the vast majority of metastases identified with AC involved microscopically small lymph nodes (< 5 mm) in our study. A fact that becomes even more relevant in the situation of neoadjuvant chemoradiotherapy, which is standard care in advanced stage of rectal cancer, leading to a smaller LN size [2, 35, 36]. Not only us, but others proved that AC does not alter LN morphology and is especially suited to retrieve small LN after neoadjuvant treatment [29]. Even though statistically not significant, additional LN metastasis found by AC in our study more frequently involved cases after neoadjuvant treatment than cases without pre-operative therapy.

Lymph node involvement is one of the key prognostic factors in CRC [5], and LN status is critical for disease staging. A correlation between the number of lymph nodes investigated and survival in CRC patients has been shown previously [35, 37–39]. Accordingly, an insufficient nodal staging may bear the risk of undiagnosed nodal metastasis, resulting in an erroneous down staging with increased risk of cancer recurrence and poorer survival outcome [40–43]. In node-positive (stage III) colon cancer, post-operative chemotherapy has become a standard treatment, and is recommended in all node-positive patients, irrespective of the number of LN metastases identified and independent of N stage (N1 and N2) [7, 44]. In contrast, neoadjuvant therapy is standard treatment in locally advanced rectal adenocarcinoma, and the role of post-operative chemotherapy in patients who received pre-operative chemoradiotherapy is controversially discussed [5, 17, 18]. In other words, the indication for post-operative chemotherapy in colon cancer is primarily guided by the presence of lymph node metastasis in the surgical resection, whereas the decision regarding neoadjuvant therapy in rectal cancer is based on pre-operative clinical staging, and post-operative therapy is often not required in rectal cancer. Thus, pathologic lymph node staging has a higher value in colon cancer with a more immediate impact on post-operative therapy compared to rectal cancer. However, identification of lymphatic metastases remains one of the key prognostic factors predicting disease recurrence and survival in both colon and rectal cancer.

In our study, 36 patients were erroneously considered as N0 (stage II) and converted to a nodal positive category (stage III) after AC. One third of metastases detected with AC concerned cases without pre-operative therapy. In these patients, AC may lead to an adaptation of the therapeutic concept with a potential new indication for post-operative treatment. Another 33 patients were already classified as nodal positive (stage III) before AC, and additional LN metastases identified by AC resulted in assignment to a higher nodal category, but the indication for post-operative chemotherapy was already given before AC. In these cases, acetone compression did not lead to an immediate change in the treatment concept. However, a higher number of LN metastases and a more advanced N stage are known to correlate with a poorer prognosis and a higher risk of disease recurrence [45]. Accordingly, the higher nodal category resulting from AC may determine a more intense post-treatment surveillance and better prognosis prediction in these patients.

Post-operative therapy is also considered in selected patients with node-negative (stage II) disease with additional high-risk features, such as inadequate LN sampling (i.e., number < 12) [11–13]. Thus, AC method facilitates the identification of true high-risk stage II patients, as it allows the detection of 12 or more LN in most surgical resection specimen.

An adequate nodal resection is not only crucial in terms of outcome and prognosis prediction, but also serves as an important quality control parameter in colorectal cancer surgery, and its reporting improves surgical quality management [31, 46]. In this respect, too, it is reasonable to work up the number of lymph nodes in a surgical resection as precisely as possible.

To note, acetone is a flammable substance and its storage should be away from sources of ignition or heat. In normal use, acetone is not considered to exhibit toxicity [47, 48]. However, inhalation of larger doses can cause temporary bronchial irritation, fatigue and headaches, and skin contact with acetone can cause dryness and irritation. With standard laboratory protective measures (i.e., ventilation, breathing protection, gloves, wash hands after use), such side effects are easily avoided. Overall, implementation of acetone compression in the routine of a pathology laboratory is technically practicable and does not require excessive measures and expenditure.

Effort and costs of acetone compression strongly depend on the amount of tissue. At our institute, processing 100 g fat tissue accounts for approx. 500–600 Swiss francs, including laboratory and medical expenditure. Larger surgical specimens may comprise several hundred grams of mesenteric fat, thus the application of acetone compression generates significant supplementary costs. In times of growing importance of cost-effectiveness in health system, standard pathologic workup with manual lymph node harvest will keep priority. However, if conventional preparation fails to identify the required number quantity of lymph nodes, acetone compression and related methods are justifiable options given the high impact of an accurate nodal stating on prognosis prediction and post-operative therapeutic strategy.

The main limitation in this study is that the number of manually harvested lymph nodes is relatively low in many cases (average 4.8). The primary reprocessing of resection specimens was performed by resident physicians at different stages of their training, and the success of lymph node harvest may depend on the experience of the person performing the dissection. Potentially, the opportunity of subsequent acetone compression may have led the investigators to perform the lymph node dissection only exemplary. On the other hand, this can also be considered an advantage, in the way that acetone compression is a method that reliably detects lymph nodes regardless of the examiner’s experience.

In summary, our study demonstrates that acetone compression is a straightforward and most efficacious method to achieve adequate LN sampling and identify 12 or more LN in almost all surgical specimen. AC leads to a significant gain in the number of lymph nodes evaluated. Additional metastases identified by AC improve nodal staging, with high relevance for decision upon post-operative treatment regimen and prognostic advantage in these patients.

## Abbreviations

AC: Acetone compression
CRC: Colorectal cancer
LN: Lymph node
